# The Intracellular HBV DNAs as Novel and Sensitive Biomarkers for the Clinical Diagnosis of Occult HBV Infection in HBeAg Negative Hepatocellular Carcinoma in China

**DOI:** 10.1371/journal.pone.0107162

**Published:** 2014-09-17

**Authors:** Hui Wang, Meng Fang, Xing Gu, Qiang Ji, Dongdi Li, Shu-Qun Cheng, Feng Shen, Chun-Fang Gao

**Affiliations:** 1 Department of Laboratory Medicine, Eastern Hepatobiliary Surgery Hospital, Second Military Medical University, Shanghai, PR China; 2 Research School of Chemistry, The Australian National University, Canberra ACT, Australia; 3 Department of Hepatic Surgery (VI), Eastern Hepatobiliary Surgery Hospital, Second Military Medical University, Shanghai, PR China; 4 Department of Hepatic Surgery (IV), Eastern Hepatobiliary Surgery Hospital, Second Military Medical University, Shanghai, PR China; Johnson & Johnson Medical, China

## Abstract

This study aimed to investigate the virological status in liver (both tumor and adjacent non-tumor tissue), the clinical features and the contribution of occult HBV infection (OBI) to postoperative prognosis in HBeAg-negative(−) hepatocellular carcinoma (HCC) patients in China. Using quantitative TaqMan fluorescent real-time PCR assays, HBV covalently closed circular DNA (cccDNA) and total DNA (tDNA) were both quantified in 11 (HBsAg(−)) and 57 (HBsAg-positive(+)) pairs of tumor tissue (TT) and adjacent non-tumor tissue (ANTT) obtained from HBeAg(−) HCC patients who received no antiviral treatment and were negative for anti-HCV before surgical treatment. Of 11 HBsAg(−) patients, 36% were with HBsAb(+) HBeAb(+) HBcAb(+). However, only 9% of the HBsAg(−) patients were HBsAb(−) HBeAb(+) HBcAb(+), which accounted for the majority (93%) in the HBsAg(+) group. TT and ANTT HBV tDNAs in 11 HCC patients with HBsAg (−) and HBeAg (−) were all detectable. HBV cccDNA and tDNA were all lower in the HBsAg(−) group than those in the HBsAg(+) group. By Kaplan-Meier analysis, patients with OBI were associated with a lower risk of cirrhosis and better overall survival (OS). The intracellular HBV DNAs, such as HBV cccDNA and tDNA are valuable biological markers for the diagnosis of occult HBV infection in HCC patients. This would assist the clinical implementation of a more personalized therapy for viral re-activation control and improve the survival rate of OBI patients.

## Introduction

Hepatocellular carcinoma (HCC) is the third most common cause of cancer death worldwide [1], [2]. The major risk factor for the development of HCC is hepatitis B virus (HBV) infection [3], [4]. A peculiar aspect of chronic HBV infection is the persistence of HBV genomes in the absence of serum HBs antigen (HBsAg), so called ‘occult’ infection. The geographic distribution of occult HBV infection (OBI) is associated with the prevalence of HBV infection and its prevalence is high in HCC populations [5].

OBI can occur not only in individuals with anti-HBs and/or anti-HBc antibodies but also in those who are negative for HBV markers [5], [6]. The seronegativity in these OBI patients may be caused by naturally occurring mutants of HBV, which alters either the immunoreactivity of various HBV proteins or the quantity of serum HBsAg [7]. The individuals with OBI usually exhibit lower levels of viremia [8]. A decrease in HBV viral load as well as replication and various relevant mutations have been implicated in the explanation of HBsAg-negative (−) [9]. Several previous studies have reported the existence of HBV DNA in liver tissues of HBsAg-negative patients [10], [11], [12], [13] and the OBI significantly correlated with cirrhosis in chronic hepatitis C virus (HCV) carriers [14], [15], [16]. OBI is a worldwide diffused entity, evidence showed that this condition might be potentially oncogenetic [5], [17]. However, in those HCC patients with HBsAg and HBeAg negative, the virologic status and the clinical features of OBI are still not thoroughly studied.

HBV covalently closed circular DNA (cccDNA) is an important intermediate in the life cycle of HBV, from which the HBV pregenomic RNA and all HBV messenger RNA transcripts originate [13]. Although the level of HBV replication in those HCC patients with HBsAg and HBeAg negative is low, little is known about the level of HBV covalently closed circular DNA (cccDNA) and total DNA (tDNA) in paired tumor tissues (TT) and adjacent non-tumor tissues (ANTT) in chronic Hepatitits B (CHB) endemic areas, such as China.

We therefore conducted a prospective study. The primary aim was to investigate the virologic status in the liver (both TT and ANTT) among these HCC patients with HBsAg (−) and HBeAg (−). The second aim was to determine the clinical features and the contribution of occult HBV infection (OBI) to postoperative prognosis for HCC patients with HBsAg (−) and HBeAg (−) in China.

## Materials and Methods

### Patients and samples

This study included a HBsAg-negative group (n = 11) and a HBsAg-positive (+) group (n = 57) of HCC patients with HBeAg (−) (between March 2007 and May 2009) who received no antiviral treatment and were negative for anti-HCV before surgical resection at the Shanghai Eastern Hepatobiliary Surgery Hospital (EHBH) in Shanghai, China. The study was approved by the Chinese Ethics Committee of Human Resources at the Second Military Medical University. All study participants provided written informed consent.

The inclusion criteria were patients with no evidence of hepatitis C virus (HCV) or hepatitis D virus (HDV) co-infection; no previous antiviral treatment; complete resection of tumor with sufficient safety margin (R0) and histologically proven HCC.

1. The HBsAg-negative group: HBsAg-negative and HBeAg-negative for at least 6 months, undetectable serum HBV DNA.
2. The HBsAg-positive group: HBsAg-positive and HBeAg-negative for at least 6 months.

The exclusion criteria included a history of liver transplantation and other malignancies, tumors of uncertain origin, metastatic liver cancer, autoimmune liver diseases, drug-related liver diseases, alcoholic hepatitis and other causes of chronic liver diseases (such as HCV, HDV, HEV, HIV) diagnosed before enrollment.

Details of patient clinical diagnosis, follow up are included in File S1.

### Quantitation of HBV cccDNA and total DNA (tDNA) in tissues

Viral DNAs in frozen tissues were extracted using the QIAamp DNA Mini kit (QIAGEN GmbH, Hilden, Germany). HBV cccDNA and tDNA were detected using real-time polymerase chain reaction (PCR) with TaqMan fluorescent probes (Fosun Diagnostics, Shanghai, China) according to the method described by Bettina et al. with a slight modification [18]. The extracted DNA samples were treated with plasmid DNA-safe ATP-dependent enzyme (Epicentre, Madison, WI). Real-time PCR was performed on an ABI 7500 (Life Technologies Corporation, Foster City, CA) using a 50 µl reaction volume containing 20 ng of DNA (for cccDNA quantification, a volume equivalent to 20 ng prior to DNase treatment), 2.5 mM MgCl_2_, 0.5 µM of forward and reverse primers, and a 0.4 µM probe. Forward and reverse primers were F1 and R1 for cccDNA amplification, respectively and F2 and R2 for total intrahepatic HBV DNA amplification, respectively. TaqMan probes were TaqP1 for cccDNA quantification and TaqP2 (Table S1) for total intrahepatic HBV DNA quantification. GAPDH, a single copy housekeeping gene present in human was used in the real-time PCR as a control to estimate the number of cells represented in each PCR reaction. Serial dilutions of genomic DNAs were used as standards to quantitate GAPDH DNA from liver tissues. The results of cccDNA and tDNA were normalized to copies/10^6^ cells.

### Statistical analysis

All statistical analyses were two sided and performed using SPSS 17.0 for Windows (SPSS Inc., Chicago, IL). A *P* value of <0.05 was considered as statistically significant. Details are included in File S1.

## Results

### Immunological characteristics of HCC patients

The immunological characteristics of HBeAg(−) HCC patients are summarized in Table 1. There was significant difference between the HBsAg(−) and the HBsAg(+) group (P<0.001). Of 11 patients in the HBsAg(−) group, 36% were with HBsAb (+) HBeAb (+) HBcAb (+). The patients with HBsAb (+) HBeAb (−) HBcAb (+), HBsAb (−) HBeAb (−) HBcAb (+) and HBsAb (−) HBeAb (−) HBcAb (−) respectively accounted for 18%. However, the percentage of patients with HBsAb (−) HBeAb (+) HBcAb (+) is significantly higher in HBsAg (+) group than in HBsAg (−) one.

**Table 1:** Immunological characteristics of HBeAg-negative HCC patients.

Characteristic	HBsAg (−) and HBeAg (−)	HBsAg (+) and HBeAg (−)	*P* value
	No. of patients (%)	No. of patients (%)	
**HBsAb (+) HBeAb (+) HBcAb (+)**	4 (36%)	1 (2%)	P<0.001
**HBsAb (+) HBeAb (−) HBcAb (+)**	2 (18%)	0	
**HBsAb (−) HBeAb (+) HBcAb (+)**	1 (9%)	53 (93%)	
**HBsAb (−) HBeAb (−) HBcAb (+)**	2 (18%)	3 (5%)	
**HBsAb (−) HBeAb (−) HBcAb (−)**	2 (18%)	0	
**Total**	11	57	

In the HBsAg(−) group, there were 6 HBsAb(+) patients (54%) and 5 HBsAb(−) patients (46%), 5 HBeAb(+) patients (46%) and 6 HBeAb(−) patients (54%), and 9 HBcAb(+) patients (82%) and 2 HBcAb(−) patients (18%). However, in the HBsAg(+) group, there were only 1 patients (2%) with HBsAb(+) and 56 patients (98%) with HBsAb(−),54 patients (95%) with HBeAb(+) and 3 patients (5%) with HBeAb(−), and 57 patients (100%) with HBcAb(+).

### Intrahepatic HBV DNAs in 11 HCC patients with HBsAg (−) and HBeAg (−) were all detectable

The paired TT/ANTT of HCC patients with HBeAg (−) were stratified into different group (A–E) according to the immunological characteristics, and examined for intracellular HBV cccDNA and tDNA levels (Table 2). Interestingly, in the HBsAg(−) group, all HBV tDNAs were detectable, and 3 TT and 5 ANTT cccDNAs were undetectable. Among the HBsAg(+) group, cccDNA was undetectable in one TT sample. The relationship between serum HBV DNA and cccDNA were shown in Table S2. Both the TT and the ANTT cccDNA made up a smaller portion of the tDNA in the HBsAg(−) group than those in the HBsAg(+) group, other than those patients with HBsAb (+) HBeAb (+) HBcAb (+).

**Table 2:** Quantification of intrahepatic HBV DNAs in HCC patients.

Characteristic	Group	HBsAb	HBeAb	HBcAb	No. of patients (%)	TT (log_10_ copies/10^6^ cells)		ANTT (log_10_ copies/10^6^ cells)		TT ratio (%)	ANTT ratio (%)
						cccDNA	HBV tDNA	cccDNA	HBV tDNA		
HBsAg (−) and HBeAg (−)	A	+	+	+	4 (36%)	3.05±0.53	4.78±1.65	3.29±0.66*	4.83±2.03	11.96±17.09	0.56±0.67
	B	+	−	+	2 (18%)	2.33±0.01	5.62±0.16	2.67±0.56	6.07±0.55	0.05±0.02	0.04±0.01
	C	−	+	+	1 (9%)	1.45	5.59	undetectable	1.64	0.01	undetectable
	D	−	−	+	2 (18%)	6.07*	5.22±5.06	undetectable	2.54±0.15	0.19*	undetectable
	E	−	−	−	2 (18%)	undetectable	2.65±0.01	4.86*	4.81±2.95	undetectable	0.92*
Total					11 (100%)						
HBsAg (+) and HBeAg (−)	A	+	+	+	1 (2%)	3.82	5.17	3.75	6.83	4.45	0.08
	B	+	−	+	0						
	C	−	+	+	53 (93%)	4.57±1.67	6.62±1.42	4.86±1.16	6.95±0.88	2.73±4.45	1.97±2.76
	D	−	−	+	3 (5%)	6.99±0.07*	6.43±3.54	4.70±1.43	6.61±0.61	4.74±4.71*	2.80±3.34
	E	−	−	−	0						
Total					57 (100%)						

Abbreviations: HBV, hepatitis B virus; cccDNA, covalently closed circular DNA; tDNA, total DNA; TT, tumor tissue; ANTT, adjacent non-tumor tissue.

“+” and “−” indicate positive and negative detection.

* indicate the concentration of one sample was undetectable.

**Figure 1:**
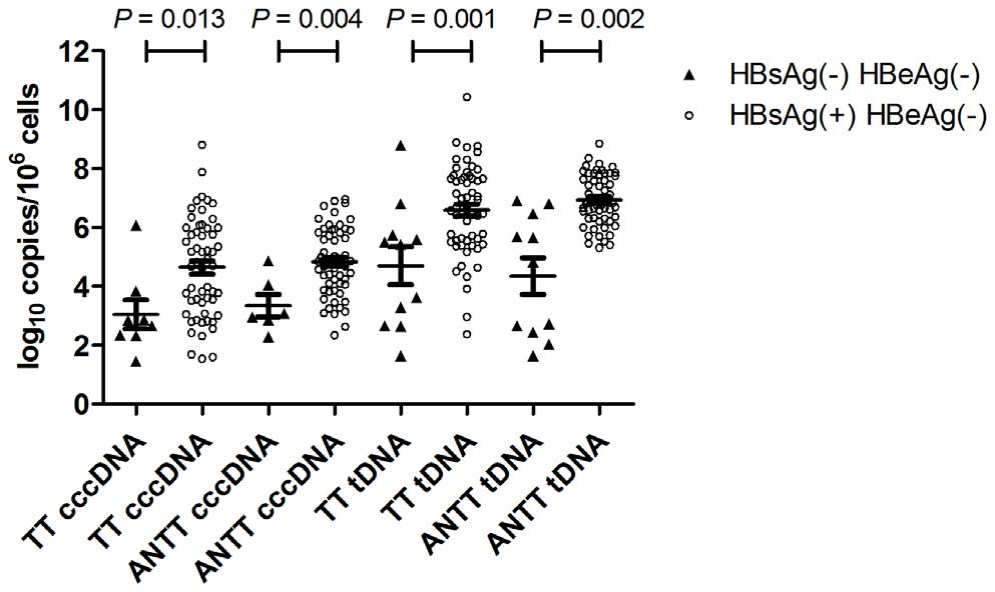
TT/ANTT tDNA and cccDNA are associated with HBsAg status in HBeAg (−) patients.

The difference in TT/ANTT HBV cccDNA and tDNA between the HBsAg(−) group and the HBsAg(+) one were shown in Figure 1. The HBV cccDNA was significantly lower in the HBsAg(−) group than in the HBsAg(+) group (TT: 3.05±1.39 vs. 4.64±1.69 log_10_ copies/10^6^ cells, *P* = 0.013; ANTT: 3.35±0.94 vs. 4.83±1.16 log_10_ copies/10^6^ cells, *P* = 0.004). Similarly, the HBV tDNA was also significantly lower in the HBsAg(−) group than in the HBsAg(+) group (TT: 4.70±2.13 vs. 6.59±1.53 log_10_ copies/10^6^ cells, *P* = 0.001; ANTT: 4.34±2.06 vs. 6.93±0.86 log_10_ copies/10^6^ cells, *P* = 0.002). However, no statistical significant difference in TT/ANTT ratios was observed between the HBsAg(−) and the HBsAg(+) group (TT: 6.02±12.87 vs. 2.83±4.40%, *P* = 0.509; ANTT: 0.45±0.55 vs. 1.98±2.75%, *P* = 0.180).

### Occult HBV infection was associated with a lower risk of cirrhosis and better overall survival

Eleven Patients with occult HBV infection had higher albumin (ALB), well-differentiated tumors (E-S grades I and II) and a lower risk to develop cirrhosis (Table 3). However, the other demographic and clinicopathologic characteristics were not significantly different between the two groups.

**Table 3:** Occult HBV infection was associated with the cirrhosis and E-S grade.

Demographic or Characteristic	HBsAg (+) and HBeAg (−) (n = 57)			HBsAg (−) and HBeAg (−) (n = 11)			*P* value
	No. of patients		%	No. of patients		%	
Sex							NS(0.360)
Male	48		84	8		73	
Female	9		16	3		27	
Age, years							NS(0.179)
Median		51			58		
Range		34–70			36–75		
AFP, ng/mL							NS(0.065)
Median		119			4		
Range		1.9->1210			1.4->1210		
CEA, ng/mL							NS(0.451)
Median		2.6			2		
Range		0.4–33.7			0.5–7.8		
CA19-9, U/mL							NS(0.779)
Median		26			13		
Range		0.6–295.5			6.7–259.9		
TBIL, µmol/L							NS(0.834)
Median		14			14		
Range		6.4–64.7			8–40.2		
ALB, g/L							0.041
Median		41			44		
Range		34.6–49.9			39.4–49.4		
ALT (IU/L)							NS(0.058)
Median		44			20		
Range		12.8–360.5			5.5–48.1		
GGT, U/L							NS(0.388)
Median		77			55		
Range		23–843			10–355		
Platelet, 10^9^/L							NS(0.913)
Median		151			##		
Range		51–382			56–284		
PT (INR)							NS(0.473)
Median		1			1		
Range		0.86–1.9			0.93–1.13		
Creatinine, µmol/L							NS(0.247)
Median		70			70		
Range		40–94			50–110		
Tumor Size, cm							NS(0.075)
Median		7.9			5		
Range		2.4–20			1.46–11.3		
Tumor Number							NS(0.568)
Single	42		74	9		82	
Multiple	15		26	2		18	
Satellite nodules							NS(0.549)
Yes	15		26	2		18	
No	41		72	9		82	
Liver cirrhosis							0.008
Yes	47		83	5		46	
No	10		18	6		55	
Tumor capsular invasion							NS(0.272)
Yes	37		65	9		82	
No	20		35	2		18	
Macrovascular invasion							NS(0.257)
Yes	14		25	1		9	
No	43		75	10		91	
E-S grade							0.015
I–II	15		26	7		64	
III–IV	42		74	4		36	
TNM stage							
I–II	30		53	8		73	NS(0.219)
III–IV	27		47	3		27	
Serum HBV DNA (log_10_ IU/mL)							<0.001
Median		4.7			undetectable		
Range		3–7.62			undetectable		
Undetectable	4		7	11		100	
detectable	53		93	0		0	

Abbreviations: NS, not significant; AFP, α-fetoprotein; ALB, albumin; CEA, carcinoembryonic antigen; GGT, γ-glutamyltransferase; TBIL, total bilirubin; ALT, alanine aminotransferase; PT, prothrombin time; E-S grade, Edmonson-Steiner grade; HBV, hepatitis B virus; HBsAg, hepatitis B sruface antigen; HBeAg, hepatitis B e antigen; HCC, hepatocellular carcinoma.

Serum HBV DNA of 11 patients with HBsAg (−) and HBeAg (−) were all less than 10^3^ IU/mL.

**Figure 2:**
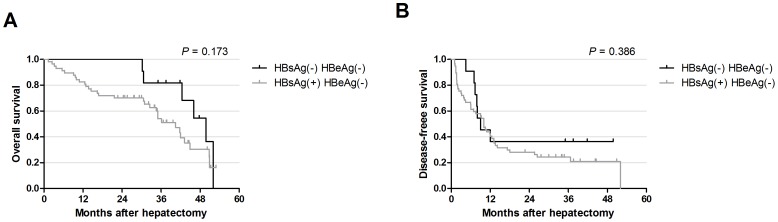
The survival of OBI patients after liver resection. Overall survival curve **A** and disease-free survival curve **B** stratified by HBsAg were constructed using Kaplan-Meier method.

By Kaplan-Meier analysis, although patients with OBI did not differ significantly in overall survival (OS) and disease-free survival (DFS) (*P* = 0.173 and *P* = 0.386, Fig. 2A, 2B), patients with OBI showed lower mortality rates at 1-, 2- and 3-years after resection.

## Discussion

HCC is one of the most common cancers worldwide, and its incidence appears to be increasing [1], [16]. Most cases of hepatocellular carcinoma (80%) arise in eastern Asia and sub-Saharan Africa, where chronic infection with HBV is the dominant risk factor [1]. However, this malignancy is not only mainly related to an overt (HBsAg positive) HBV infection, but also linked with occult HBV infection (HBsAg negative) [5]. The long-lasting persistence of HBV genomes in the liver (with detectable or undetectable HBV DNA in the serum of individuals testing negative for HBsAg) is termed OBI [17]. Our study based on the quantitative TaqMan fluorescent real-time PCR assay provides valuable information on the clinical and virological features of HCC patients with both HBsAg and HBeAg negative.

In our study, HBV tDNAs in 11 HCC patients with HBsAg(−) and HBeAg(−) were all detectable in TT or ANTT at the time of surgical resection. There were approximately half of these patients with HBsAb(+) (54%) and with HBeAb(+) (46%), and 82% of patients with HBcAb(+) in the HBsAg(−) and HBeAg(−) group. We confirmed that HBV could persist in liver after the disappearance of HBsAg in individuals with previous exposure to the virus, retaining the serological footprint of HBcAb positivity with such a virologic status [19]. But there were 2 cases with all serological markers negative (HBsAg, HBeAg, HBsAb, HBeAb and HBcAb), in which had detectable HBV tDNA in TT/ANTT. Previous studies have revealed that the HCC patients with OBI who are HBsAg-negative but positive for HBcAb are at risk of HBV reactivation after undergoing chemotherapy or immunosuppressive therapy [20], [21], [22]. Thus, it is very important to monitor HBV DNA levels regularly to achieve the early administration of antiviral or antineoplastic drugs before the onset of ALT elevation, however, the optimum testing frequency and noninvasive detection technology of HBV DNA in occult HBV carriers will need additional study.

To clarify the virological characteristics of HBV, we detected the cccDNA levels in cancerous tissues and non-cancerous tissues. cccDNA does not take part in replication directly, because it is maintained as a stable pool inside the hepatocyte nuclei [23]. We found that the levels of cccDNA and tDNA in cancerous tissues and non-cancerous tissues were significantly lower in the HBsAg(−) group than in the HBsAg(+) group. Both TT and ANTT cccDNA made up a smaller portion of the tDNA in the HBsAg-negative group, other than the tumor tissue ratio in the type of HBsAb (+) HBeAb (+) HBcAb (+). It is likely that OBI reactivates with the development of an immunosuppressive status.

Although the cause of OBI reactivation is yet to be understood, it is necessary to consider the following factors: the host's immune surveillance, restored virus, the impact of liver cancer cells and coinfection of other types of HBV. (1) Virus factors: In most cases there is no change in the α determinant that could explain the lack of HBsAg detection [24], [25], [26]. Although in a few cases (10%) the lack of HBsAg detection is due to infection with mutated viruses unrecognized by available assays (S-escape mutants) [27], [28], [29], the typical OBI is related to strongly suppressed HBV replication and the cause of HBV suppression is yet to be understood [17]. (2) Host factors: The genetic differences between individuals can lead to different immunological environment. An *in vitro* study showed that occult viral isolates could fully restore replication, transcription, and protein synthesis abilities once the viruses are taken out of the host liver microenvironment [30]. However, the association between host genomic variation and virus replication suppression needs to be investigated. (3) Coinfection. Because the exclusion criteria in our study included a history of other causes of chronic liver diseases (such as HCV, HDV, HEV, HIV) diagnosed before enrollment, there is no evidence of direct effects of infection of other types of HBV. Further studies are required to determine the characteristics of the reactivated viruses in HBsAg and HBeAg negative but HBsAb(+), HBeAb(+) and HBcAb(+) occult HBV carriers.

In this study, we found that patients with occult HBV infection are less likely to develop cirrhosis and had better overall survival. This observation strongly supported the possible contribution of OBI to the establishment of cirrhosis and the possible direct or indirect role in the development of HCC. In patients with diagnosable/detectable low-grade HBV replication, the virus retains its pro-oncogenic properties [31] [17]. Therefore, the mechanisms leading to HCC in occult HBV carriers seem to be similar to those in overt cases.

Our study has limitations. Firstly, samples were from a single department and the size was limited. In future studies, larger sample size would be preferred in order to validate the findings shown in this study. Besides, studies should continue to functionally characterize viral mutations and the relevant viral genes.

In summary, our findings suggest that the intracellular HBV DNAs, such as HBV cccDNA and tDNA are valuable biological markers for the diagnosis of occult HBV infection in HCC patitents. This would assist the clinical implementation of a more personalized therapy for viral re-activation control and improve the survival rate of OBI patients.

## Supporting Information

Table S1 **Sequences of primers used in the study for cccDNA and tDNA.**
(DOC)

Table S2 **Correlations among HBV DNAs in the HBsAg-positive group.**
(DOCX)

File S1 **Supplemental Materials and Methods.**
(DOC)
