# Supplementary material for: The Intracellular HBV DNAs as Novel and Sensitive Biomarkers for the Clinical Diagnosis of Occult HBV Infection in HBeAg Negative Hepatocellular Carcinoma in China
Source: PLoS One. 2014 Sep 17;9(9):e107162. doi: 10.1371/journal.pone.0107162 (PMC4167849; doi:10.1371/journal.pone.0107162)
Supplement: Table S1 — Sequences of primers used in the study for cccDNA and tDNA. (DOC) [file pone.0107162.s001.doc]

**Table S1**. Sequences of primers used in the study for cccDNA and tDNA.

| Name | Position | Direction | Sequence (5'-3') |
| --- | --- | --- | --- |
| F1 | 1549-1565 | Forward | TCCCCGTCTGTGCCTTC |
| R1 | 1902-1887 | Reverse | CCCCAAAGCCACCCAA |
| F2 | 257-273 | Forward | TCGTGGTGGACTTCTCT |
| R2 | 419-406 | Reverse | GCAGGATGAAGAGG |
| TaqP 1 | - | - | FAM – ATCTGCCGGACCGTGTGC – TAMARA |
| TaqP 2 | - | - | FAM – CTCACCAACCTCCTGTCCTCCA – TAMARA |
