# Supplementary material for: The Intracellular HBV DNAs as Novel and Sensitive Biomarkers for the Clinical Diagnosis of Occult HBV Infection in HBeAg Negative Hepatocellular Carcinoma in China
Source: PLoS One. 2014 Sep 17;9(9):e107162. doi: 10.1371/journal.pone.0107162 (PMC4167849; doi:10.1371/journal.pone.0107162)
Supplement: Table S2 — Correlations among HBV DNAs in the HBsAg-positive group. (DOCX) [file pone.0107162.s002.docx]

**Table S2.** Correlations among HBV DNAs in the HBsAg-positive group

| Correlation | | | TT | |  | ANTT | |
| --- | --- | --- | --- | --- | --- | --- | --- |
|  |  |  | cccDNA | HBV tDNA |  | cccDNA | HBV tDNA |
| TT | ccc DNA | r | 1 | 0.886** |  | 0.385** | 0.254 |
| (log_10_ copies/10^6^ cells) |  | *P* |  | < 0.001 |  | 0.003 | 0.059 |
|  | HBV tDNA | r |  | 1 |  | 0.295** | 0.256 |
|  |  | *P* |  |  |  | 0.026 | 0.055 |
| ANTT | cccDNA | r |  |  |  | 1 | 0.830** |
| (log_10_ copies/10^6^ cells) |  | *P* |  |  |  |  | < 0.001 |
|  | HBV tDNA | r |  |  |  |  | 1 |
|  |  | *P* |  |  |  |  |  |
| Serum | HBV DNA | r | 0.150 | 0.152 |  | 0.506** | 0.501** |
| (log_10_ IU/mL) |  | *P* | 0.270 | 0.259 |  | < 0.001 | < 0.001 |

Abbreviations: HBV, hepatitis B virus; cccDNA, covalently closed circular DNA; tDNA, total DNA; TT, tumor tissue; ANTT, adjacent non-tumor tissue

Statistically significant differences are marked **(p < 0.01).
