# Supplementary material for: The Intracellular HBV DNAs as Novel and Sensitive Biomarkers for the Clinical Diagnosis of Occult HBV Infection in HBeAg Negative Hepatocellular Carcinoma in China
Source: PLoS One. 2014 Sep 17;9(9):e107162. doi: 10.1371/journal.pone.0107162 (PMC4167849; doi:10.1371/journal.pone.0107162)
Supplement: File S1 — Supplemental Materials and Methods. (DOC) [file pone.0107162.s003.doc]

# Supplemental Materials and Methods

Patients and Samples

The clinical diagnosis of HCC was in line with the criteria of the American Association for the Study of Liver Diseases (AASLD).[1] Histopathological study of surgical specimens was carried out independently by three pathologists who came to a consensus by discussion if there was any controversy, as well as tumor characteristics including vascular invasion, histologic differentiation, satellite nodules, liver cirrhosis, and tumor capsular invasion. HCC was histologically classified using the Edmondson-Steiner classification,[2] and clinically classified using the TNM/AJCC staging systems.[3] Histologic evaluation of surgical specimens showed that all the tumors were successfully resected with sufficient safety margins.

Tumor number, largest tumor diameter (preoperative axial imaging findings), alanine aminotransferase (ALT) and a-fetoprotein (AFP).medical history and other related information were obtained from medical documentation.

Liver resection specimens included 2 slices, i.e. TT and ANTT. All tissue samples (approximately 1 cm^3^) were immediately frozen at -80°C and were used for DNA extraction. Sera collected before treatment were stored at -20°C until analysis.

Detection of Serum HBV DNA, HBsAg and HBeAg

When available, circulating serum HBV DNA, HBsAg and HBeAg prior to surgery was obtained from medical record. At our institution, serum HBV DNA was quantitated using the diagnostic kit for quantification of hepatitis B virus DNA (Kehua, Shanghai, China) on an ABI 7500 (Life Technologies Corporation, Foster City, CA). The lowest detection limit of HBV DNA in this study was 1000 IU/mL. Serum HBsAg was quantitated using the HBsAg QT assay (Roche Diagnostics GmbH, Mannheim, Germany) and other serum HBV markers (HBeAg, HBsAb, HBeAb and HBcAb) qualitatively using the Roche Diagnostics GmbH, Mannheim, Germany.

Follow-up

After discharge from hospital, the patients were followed-up once every 2 months in the first 2 years and then once every 3 months. Disease free survival (DFS) was calculated from the date of surgery to the date when HCC recurrence was first identified. Overall survival (OF) was calculated from the date of surgery to death, or to the last date of follow-up, whichever came first.

Imaging with CT or MRI, serum AFP, liver function test, and an abdominal ultrasound were performed routinely at each of the follow-up visits. A contrast-enhanced CT or MRI was performed once every 6 months, or earlier when tumor recurrence was clinically suspected. HCC recurrence/metastasis was diagnosed based on imaging findings on follow-up CT or MRI, in addition to AFP.

Statistical Analysis

The χ2 test and Fisher’s exact test was used to determine the differences in categorical variables. Continuous variables, such as intrahepatic HBV tDNA and cccDNA levels with skewed distribution were adjusted to normal distribution by transformation into logarithmic function, and then tested by the Student’s t-test, paired-samples t-test or analysis of variance, where appropriate. For linear associations, the Pearson's or Spearman’s correlation coefficients were performed.

The clinical follow-up started at the time of HCC diagnosis. Years were censored on the date of identifying HCC recurrence, death, the last date of follow-up, whichever came first. Cumulative recurrence rates and overall survival rates were estimated by the Kaplan-Meier method compared using log-rank tests and Wilcoxon tests for categorical variables.

All statistical analyses were two sided, and performed using SPSS 17.0 for Windows (SPSS Inc., Chicago, IL). A *P* value of < 0.05 was considered as statistically significant.

**References**

1. Bruix J, Sherman M (2011) Management of hepatocellular carcinoma: an update. Hepatology 53: 1020-1022.

2. EDMONDSON HA, STEINER PE (1954) Primary carcinoma of the liver: a study of 100 cases among 48,900 necropsies. Cancer 7: 462-503.

3. Wittekind C (2010) [2010 TNM system: on the 7th edition of TNM classification of malignant tumors]. Pathologe 31: 331-332.
